# Melittin-induced long non-coding RNA NONHSAT105177 inhibits proliferation and migration of pancreatic ductal adenocarcinoma

**DOI:** 10.1038/s41419-018-0965-3

**Published:** 2018-09-20

**Authors:** Xinjing Wang, Hongzhe Li, Xiongxiong Lu, Chenlei Wen, Zhen Huo, Minmin Shi, Xiaomei Tang, Hao Chen, Chenghong Peng, Yuan Fang, Xiaxing Deng, Baiyong Shen

**Affiliations:** 1 Research Institute of Pancreatic Disease, Ruijin Hospital, School of Medicine, Shanghai Jiao Tong University, Shanghai, China; 2 Pancreatic Disease Centre, Ruijin Hospital, School of Medicine, Shanghai Jiao Tong University, Shanghai, China; 3 Shanghai Institute of Digestive Surgery, Ruijin Hospital, School of Medicine, Shanghai Jiao Tong University, Shanghai, China

## Abstract

Long non-coding RNAs (lncRNAs) play crucial roles in the pathogenesis of pancreatic ductal adenocarcinoma (PDAC). Previously, we found that melittin treatment suppressed PDAC tumor growth. However, it is unclear whether lncRNAs have any role in the melittin-induced suppression of PDAC. In this study, we used microarray data to identify 844 lncRNAs that were significantly differentially expressed in response to melittin treatment. Of these lncRNAs, we focused on the lncRNA NONHSAT105177, which had about a 22-fold increase in expression with melittin treatment. We found that melittin treatment increased NONHSAT105177 expression in PDAC cell lines but not in normal pancreatic ductal epithelial cell line. NONHSAT105177 expression was significantly lower in PDAC cancer tissues than in adjacent noncancerous tissues. Additionally, overexpression of NONHSAT105177 inhibited PDAC cell proliferation, migration, and the epithelial–mesenchymal transition (EMT), both in vitro and in vivo. Consistent with the mechanism of action of melittin, NONHSAT105177 significantly downregulated cholesterol pathway genes, including Clusterin (CLU). Moreover, we found that NONHSAT105177 trafficking was mediated by exosomes. The combined findings of our current and previous studies suggest that NONHSAT105177 mediated the melittin-induced inhibition of PDAC cell growth and metastasis, which indicated a potential target for developing new strategies.

## Introduction

Pancreatic ductal adenocarcinoma (PDAC) is a particularly lethal cancer, due to its poor sensitivity to chemotherapy. It is the leading cause of cancer death worldwide in both men and women^1^. To improve the prognosis for patients with PDAC, there is an urgent need to gain new insights into the biological characteristics of this cancer and develop novel treatment strategies.

The underlying mechanisms of PDAC have been linked to long non-coding RNAs (lncRNAs)^2,3^. The discovery of lncRNAs has been one of the most unexpected findings of the genomics era and provides a promising avenue for gaining biological insights^4,5^. LncRNAs are functionally defined as transcripts of >200 nucleotides that have no protein-coding potential. Although many lncRNAs are uniquely expressed in different tissues or specific cancer types^6–9^, the specific mechanisms of lncRNAs in PDAC pathogenesis require further investigation.

Previously, we demonstrated that a compound called melittin suppressed PDAC tumor growth by regulating the cholesterol pathway gene Clusterin (CLU)^10^. Melittin is a water-soluble cationic amphipathic 26-aa α-helical peptide that is derived from the venom of the honeybee *Apis mellifera*^11^. Melittin disrupts both the physical and chemical structure of the cell membrane, which profoundly compromises the cell permeability barrier due to cell lysis. However, it is unknown whether lncRNA has a role in melittin-induced suppression of PDAC cells.

In this study, we first analyzed lncRNA expression by microarray to identify melittin-induced lncRNA dysregulation in PDAC cell lines. Among the 844 significantly dysregulated lncRNAs, we focused on the lncRNA NONHSAT105177 to further investigate its potential role in PDAC carcinogenesis, using both in vitro and in vivo models.

## Results

### Global profiling of lncRNAs in melittin-treated SW1990 cells

**Fig. 1:**
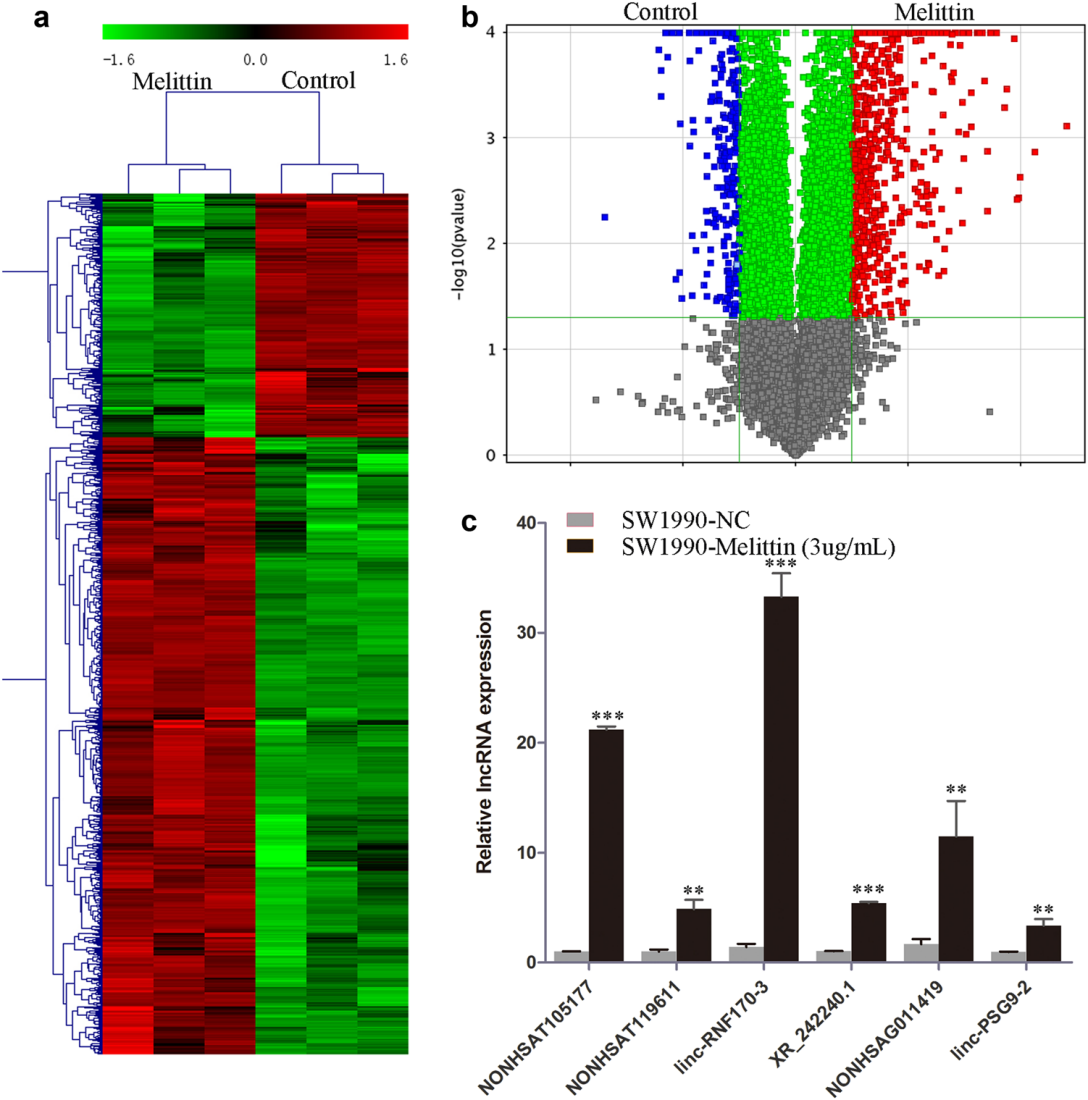
Global profiling of long non-coding RNAs (lncRNAs) in melittin-treated SW1990 cells. **a** Hierarchical clustering analysis of lncRNAs; **b** Volcano plot analysis of the microarray data for differentially expressed lncRNAs comparing the melittin-treated and control groups; **c** Quantitative reverse transcription–polymerase chain reaction (qRT-PCR) validation of differentially expressed lncRNAs. (**P < 0.01, ***P < 0.001)

In our previous study, we determined that melittin treatment reduced the viability of SW1990 cells by about 40%^10^. We used the same melittin dose (3 μg/ml) and treatment time in the current study to determine the role of lncRNAs in melittin-induced suppression of PDAC cells. To investigate the expression patterns of lncRNAs, we applied hierarchical clustering to gene expression analysis. We found that lncRNAs were differentially expressed between melittin-treated and control groups (Fig. 1a, Supplementary Table 2). In the volcano plot analysis (Fig. 1b), we identified a total of 844 significantly differently expressed lncRNAs (fold change >2; *P* < 0.05; Supplementary Table 2). Of these, 605 were significantly upregulated by melittin treatment (Supplementary Table 2). To verify the microarray results, we performed quantitative reverse transcription–polymerase chain reaction (qRT-PCR) and obtained consistent results for six selected lncRNAs (Fig. 1c).

### The lncRNA NONHSAT105177 was dysregulated in PDAC cells

**Fig. 2:**
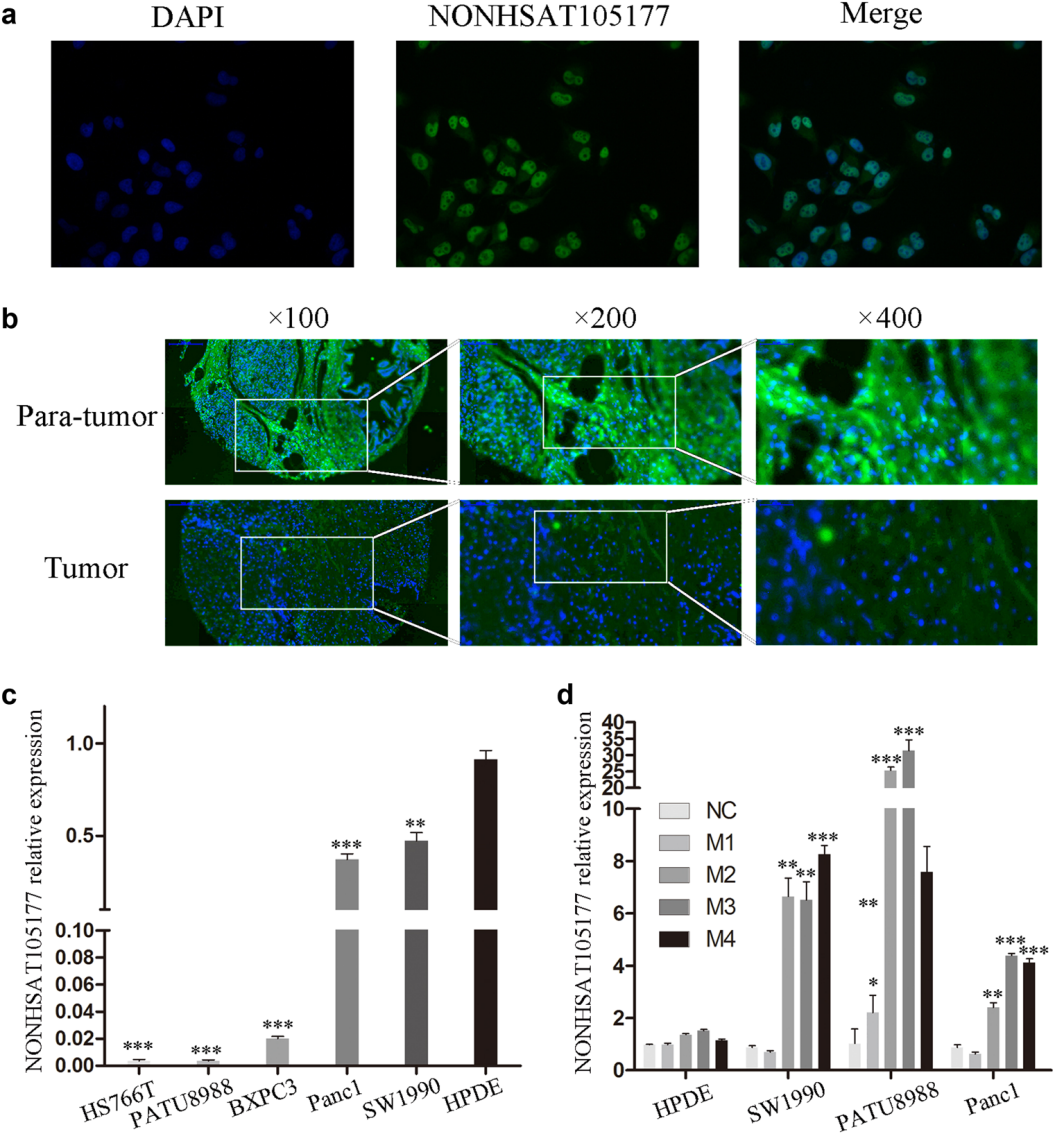
The lncRNA NONHSAT105177 was dysregulated in pancreatic ductal adenocarcinoma (PDAC) cells. **a** Fluorescence in situ hybridization (FISH) depicting NONHSAT105177 localization in PDAC cells; **b** Expression of NONHSAT105177 in paired tumor and para-tumor tissue of PDAC specimens; **c** Expression of NONHSAT105177 in the PDAC cell lines HS766T, PATU8988, BXPC3, Panc1, SW1990, and normal pancreatic ductal epithelial cell line HPDE; **d** Melittin selectively induced NONHSAT105177 expression in PDAC cells but not in normal HPDE. (*P < 0.05, **P < 0.01, ***P < 0.001)

Of the differentially expressed lncRNAs, we focused on lncRNA NONHSAT105177 for its expression level increased by about 22-fold with melittin treatment (Fig. 1c). Using fluorescence in situ hybridization (FISH), we found that NONHSAT105177 was mainly located in the nuclei of PDAC cells (Fig. 2a). Next, we further analyzed NONHSAT105177 expression in paired tumor and para-tumor tissue, finding that the expression of NONHSAT105177 was lower in the tumor tissue than in the para-tumor tissue (Fig. 2b). Additionally, we analyzed NONHSAT105177 expression in the PDAC cell lines HS766T, PATU8988, BXPC3, Panc1, and SW1990. We found that NONHSAT105177 expression was much lower in these cell lines than in the normal pancreatic ductal epithelial cell line, HPDE (Fig. 2c).

The most interesting point is that melittin treatment selectively induced NONHSAT105177 expression in PDAC cells but not in normal HPDE (Fig. 2d). Further, the induction of NONHSAT105177 in PDAC cells was dose-dependent, such that the strongest effect was observed when the concentration of melittin was ≥2 μg/ml (Fig. 2d).

### Upregulation of NONHSAT105177 suppressed the growth and migration of PDAC cells

**Fig. 3:**
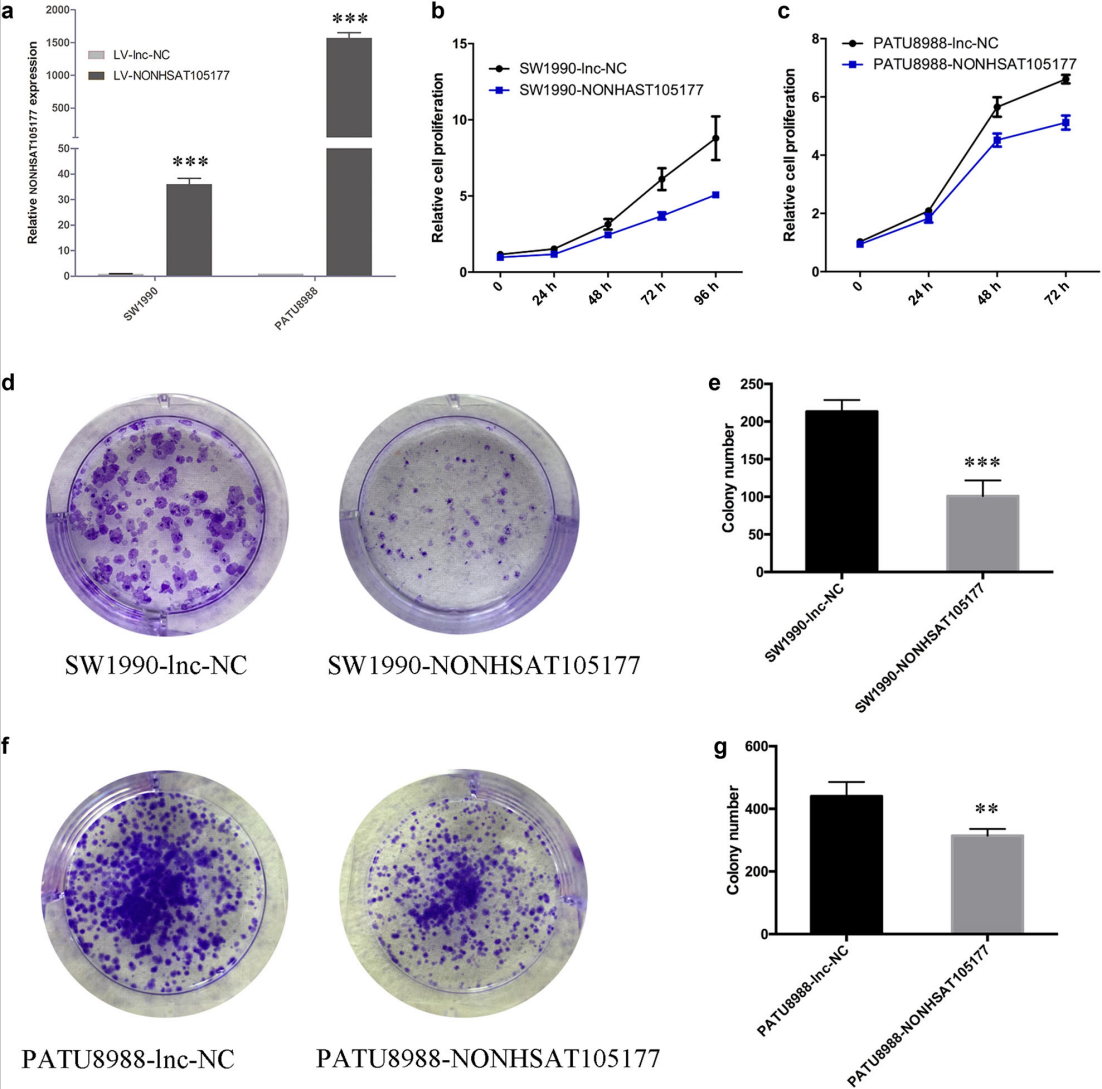
Upregulation of NONHSAT105177 suppressed the growth of pancreatic ductal adenocarcinoma (PDAC) cells. **a** qPCR quantification of NONHSAT105177 overexpression in SW1990 and PATU8988 cell lines; **b**, **c** CCK8 assay to confirm the effect of NONHSAT105177 on cell proliferation; **d**–**g** Colony-formation assay to determine the ability of cells to form colonies. (**P < 0.01, ***P < 0.001)

To further study the effects of NONHSAT105177 on PDAC cell proliferation, we established stable overexpression cell lines via a lentiviral infection of SW1990 and PATU8988 and validated upregulation of NONHSAT105177 by qPCR (Fig. 3a). Overexpression of NONHSAT105177 significantly decreased the proliferation rates of PDAC cells (Fig. 3b, c) and inhibited the colony formation of PDAC cells (Fig. 3d–g).

**Fig. 4:**
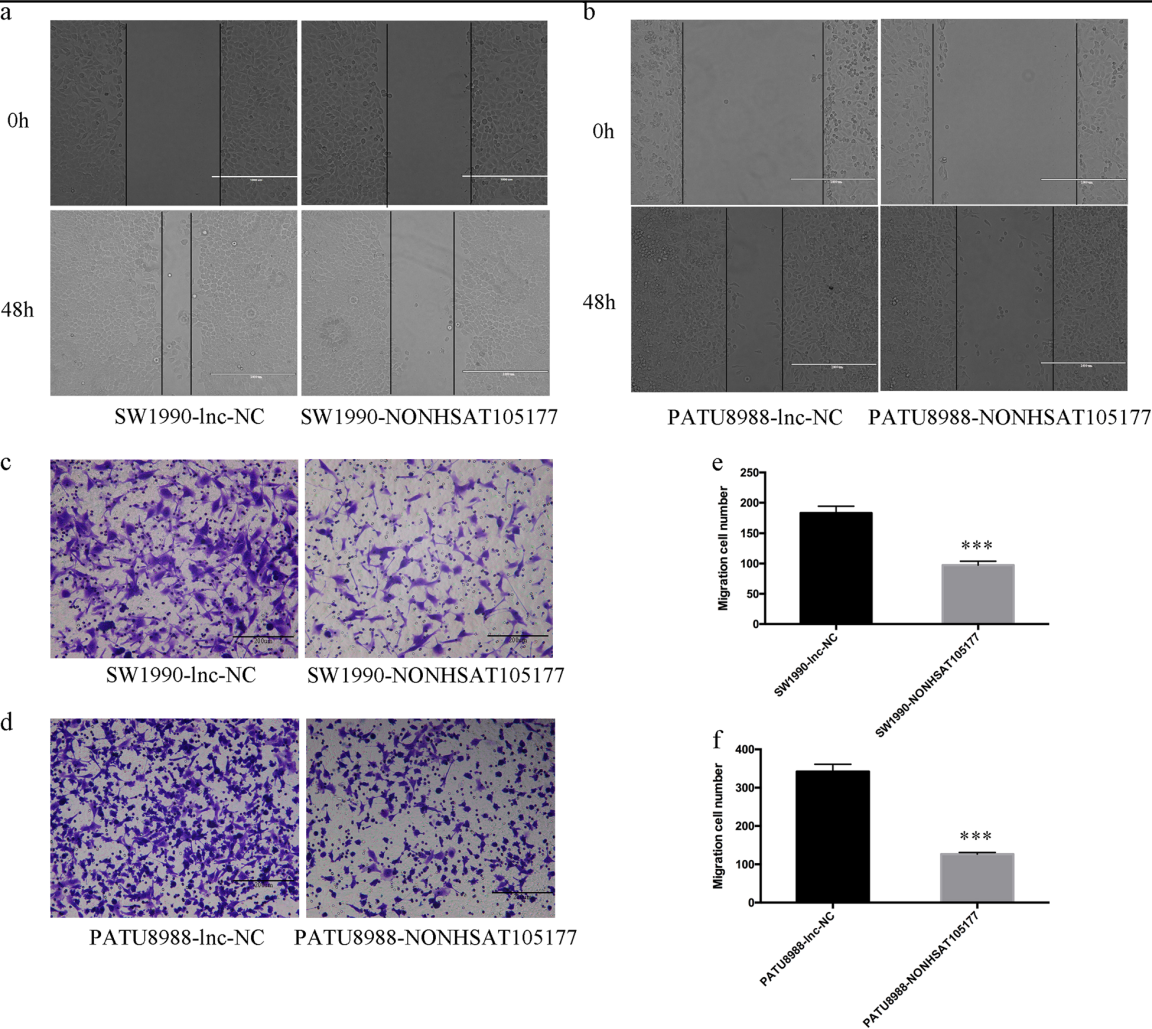
Upregulation of NONHSAT105177 suppressed the migration of pancreatic ductal adenocarcinoma (PDAC) cells. **a**–**b**. Wound healing assay to determine the effect of NONHSAT105177 overexpression on cell migration; **c**–**f** Transwell assay to determine the effect of NONHSAT105177 overexpression on cell migration. (***P < 0.001)

Our previous study reported that melittin suppressed PDAC growth^10^. In this study, we found that melittin inhibited PDAC cell migration in a dose-dependent manner (Supplementary Figure 1A-B). To explore the role of NONHSAT105177 in PDAC cell metastasis, we performed wound healing and transwell migration assays. Compared to control cells, PDAC cells overexpressing NONHSAT105177 had significantly decreased wound healing (Fig. 4a, d) and inhibited cell migration (Fig. 4e–h). This supported the role of NONHSAT105177 in inhibiting metastasis of PDAC cells.

### NONHSAT105177 inhibited epithelial–mesenchymal transition (EMT) progression in PDAC cells

**Fig. 5:**
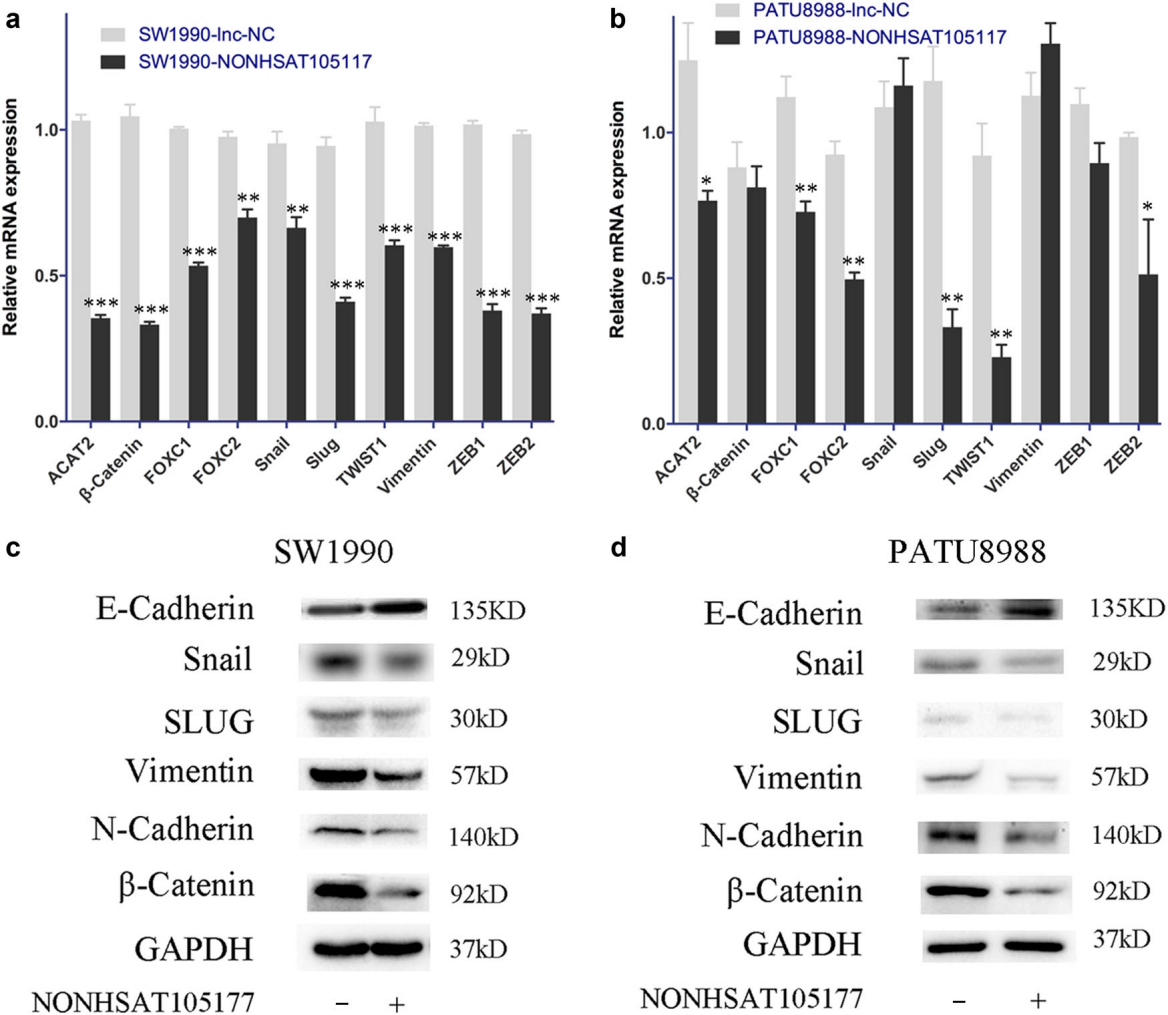
Upregulation of NONHSAT105177 inhibited epithelial–mesenchymal transition (EMT) progression in pancreatic ductal adenocarcinoma (PDAC) cells. **a**, **b** mRNA levels of EMT pathway molecules (ACAT2, β-catenin, FOXC1, FOXC2, Snail, Slug, TWIST1, Vimentin, ZEB1, and ZEB2) in response to overexpression of NONHSAT105177 in SW1990 (**a**) and PATU8988 (**b**); **c**, **d** Protein levels of EMT pathway molecules in response to overexpression of NONHSAT105177 in SW1990 (**c**) and PATU8988 (**d**). (*P < 0.05, **P < 0.01, ***P < 0.001)

EMT is thought to be one of the most important processes for acquiring metastatic potential during cancer progression^12–14^. EMT is widely acknowledged to be controlled mainly by transcription factors, such as the Snail family, that repress E-cadherin expression and increase levels of N-cadherin. We found that melittin treatment reduced protein levels of Snail and Slug, as well as the levels of the mesenchymal marker Vimentin (Supplementary Figure 1C). In contrast, melittin treatment upregulated E-cadherin expression (Supplementary Figure 1C). Furthermore, in NONHSAT105177-overexpressing PDAC cells, we observed downregulated mRNA levels of ACAT2, β-catenin, FOXC1, FOXC2, Snail, Slug, TWIST1, Vimentin, ZEB1, and ZEB2 (Fig. 5a, b). Accordingly, we saw lower protein levels of Snail, Slug, N-cadherin, Vimentin, and β-catenin in NONHSAT105177-overexpressing cells, while the expression of E-Cadherin was upregulated (Fig. 5c, d). These data suggested that NONHSAT105177 regulated the migration potential of pancreatic cancer cell lines via the canonical pathway involving EMT-inducing transcription factors.

### NONHSAT105177 overexpression downregulated the cholesterol biosynthesis pathway in PDAC cells

**Fig. 6:**
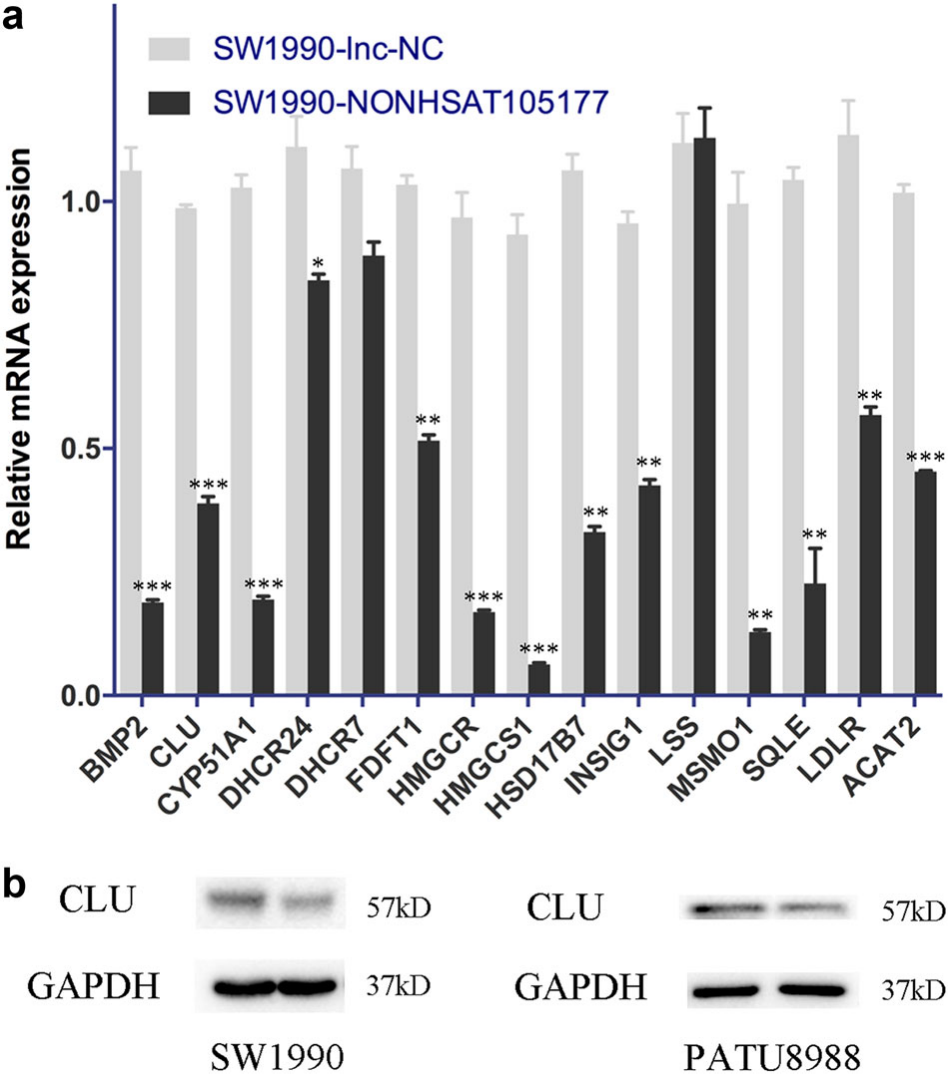
Upregulation of NONHSAT105177 downregulated cholesterol biosynthesis pathway in pancreatic ductal adenocarcinoma (PDAC) cells. **a** mRNA expression of cholesterol biosynthesis pathway molecules (BMP2, CLU, CYP51A1, DHCR24, DHCR7, FDFT1, HMGCR, HMGCS1, HSD17B7, INSIG1, LSS, MSMO1, SQLE, LDLR, and ACAT2) in response to overexpression of NONHSAT105177; **b** Protein levels of cholesterol biosynthesis pathway molecule CLU in response to overexpression of NONHSAT105177. (*P < 0.05, **P < 0.01, ***P < 0.001)

Aberrant activation of cholesterol biosynthesis has been observed in different tumor types, including PDAC^15,16^. Previously, we showed that melittin inhibits the growth of PDAC cells by downregulating the CLU cholesterol biosynthesis pathway^10^. To test the role of NONHSAT105177 in cholesterol biosynthesis, we first examined the expression levels of cholesterol pathway genes in NONHSAT105177-overexpressing cells. Interestingly, NONHSAT105177 overexpression decreased the mRNA levels of many cholesterol biosynthesis genes (BMP2, CLU, CYP51A1, DHCR24, DHCR7, FDFT1, HMGCR, HMGCS1, HSD17B7, INSIG1, LSS, MSMO1, SQLE, LDLR, and ACAT2) (Fig. 6a), which is consistent with the mechanism of action of melittin. Furthermore, CLU protein levels were also reduced by NONHSAT105177 overexpression (Fig. 6b, c). Taken together, these data indicated that melittin inhibited PDAC by targeting CLU, in a manner mediated by NONHSAT105177 induction.

### Trafficking of NONHSAT105177 was mediated by exosomes

**Fig. 7:**
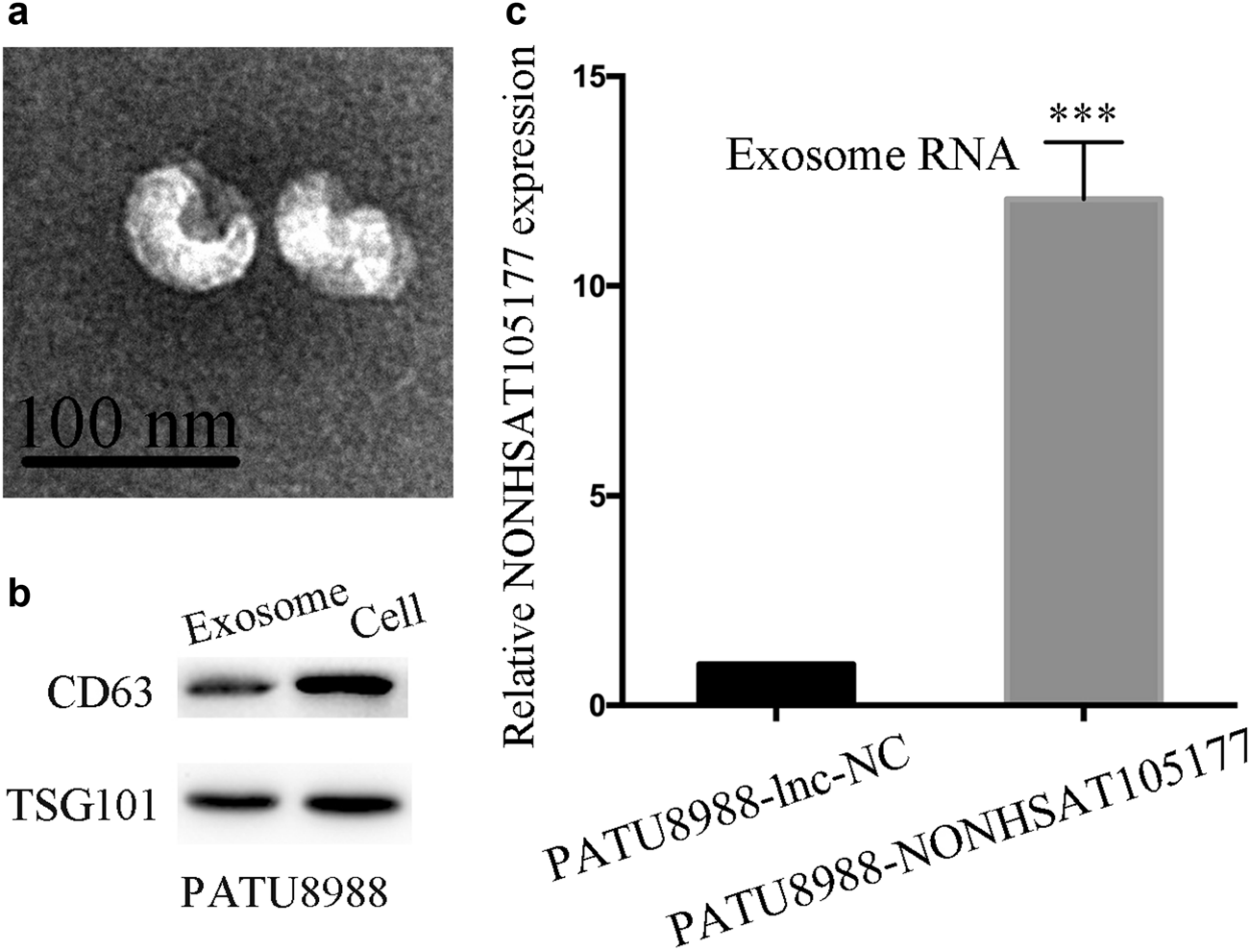
Trafficking of NONHSAT105177 was mediated by exosomes. **a** Scanning electron microscopic images of purified exosomes; **b** Western blot analysis showing the presence of CD63 and TSG101 in exosomes derived from the conditioned medium of PATU8988 cells; **c** NONHSAT105177 expression in exosomes secreted by PATU8988-NONHSAT105177 and the contrasting cell line. (***P < 0.001)

Exosomes are secreted by multiple cells and participate in intercellular communication by transmitting cargoes like proteins and nucleic acids. Recently, some studies have proposed that exosomes from stromal cells may affect therapeutic response via transfer of ncRNAs^17,18^. Here we purified tumor-derived exosomes from the supernatant of PATU8988 cells that were stably transfected with NONHSAT105177 lentivirus. The purified exosomes were examined by scanning electron microscopy, which showed rounded particles that ranged from 50 to 100 nm in diameter that are typical of exosomes (Fig. 7a). We further confirmed the presence of the known exosome markers CD63 and TSG101 by western blot analysis (Fig. 7b). Interestingly, the expression of NONHSAT105177 in exosomes differed between PATU8988-NONHSAT105177 and PATU8988-lnc-NC cells. Exosomes secreted by PATU8988-NONHSAT105177 cells had significantly higher expression of NONHSAT105177 than the contrast (Fig. 7c).

### NONHSAT105177 restricted tumor growth in xenograft mouse model

**Fig. 8:**
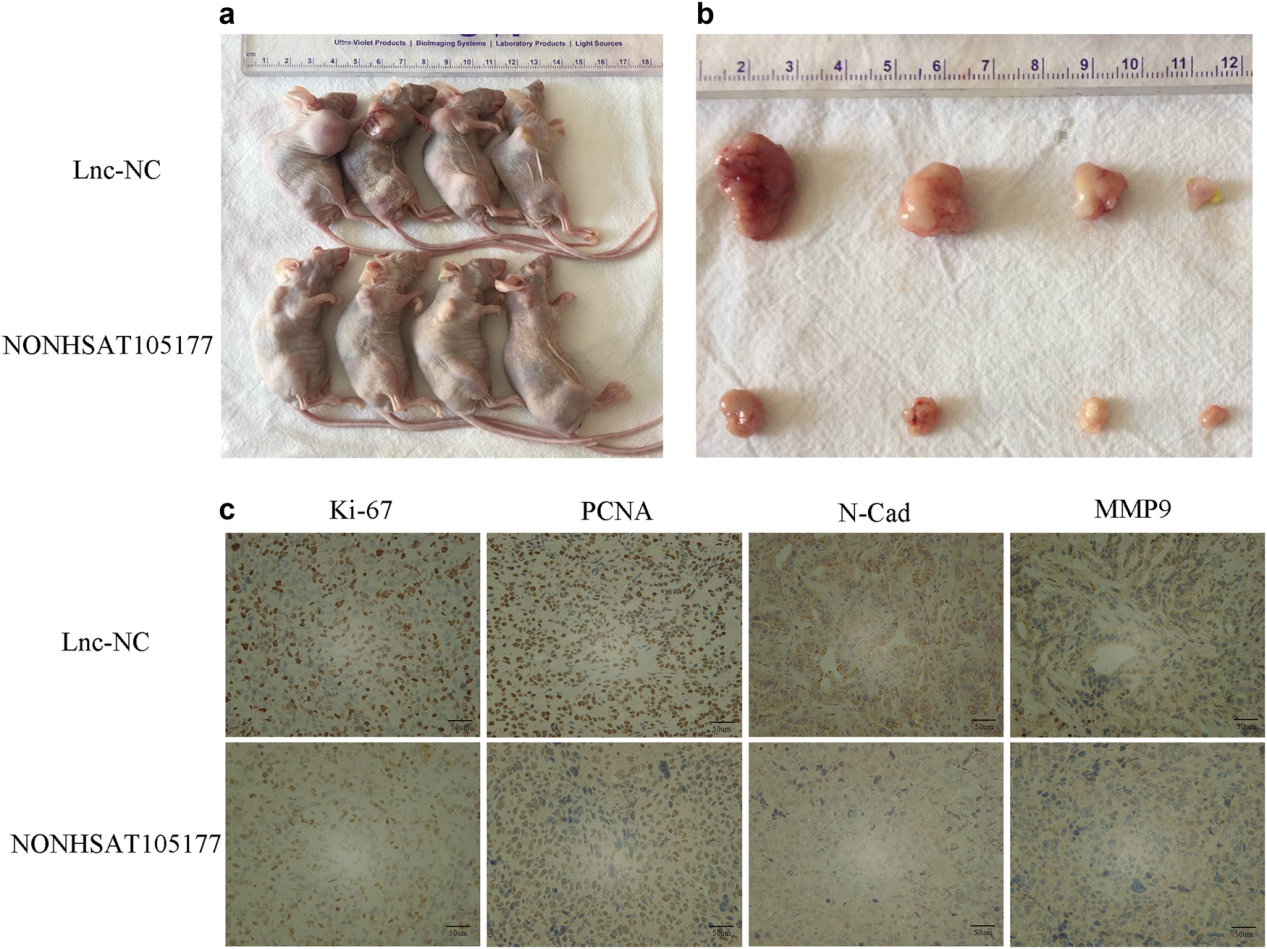
Upregulation of NONHSAT105177 restricted tumor growth in xenograft mouse model. **a** Xenograft mice from each treatment group. **b** Tumors harvested from each treatment group. **c** Immunohistochemistry for Ki-67, PCNA, N-Cad, and MMP9 expression in tumor sections

Given that NONHSAT105177 overexpression inhibited PDAC cells in vitro, we further evaluated NONHSAT105177 suppression of tumor growth in a xenograft mouse model. We subcutaneously injected the PATU8988-NONHSAT105177 and PATU8988-lnc-NC cells into nude mice and monitored the growth of the xenograft. Tumor volume in the NONHSAT105177-overexpressing group was less than that of the control group (Fig. 8a, b). Additionally, we examined the proliferative index of Ki-67 and proliferating cell nuclear antigen (PCNA) by immunohistochemistry (IHC) staining of the tumor graft, finding that the frequency of cells positive for Ki-67 and PCNA was significantly lower (*P* < 0.01) in the PATU8988-NONHSAT105177 tumor than in the PATU8988-lnc-NC tumor. Furthermore, the protein levels of two metastasis-related markers, N-cadherin and MMP9, were lower in the NONHSAT105177-overexpressing group than in the control group (Fig. 8c).

## Discussion

In our previous study, we found that melittin suppressed tumor growth and promoted gemcitabine sensitivity in PDAC by downregulating cholesterol biosynthesis pathway^10^. That study shed light on the significance of cholesterol biosynthesis in melittin-mediated control of PDAC. In the present study, we further investigated the regulatory roles of melittin and lncRNA in PDAC. We first performed lncRNA microarray analysis and discovered a total of 844 lncRNAs with expression that was significantly altered and had a distinct expression profile change in response to melittin treatment in SW1990 cells, which was validated by qPCR. Thus lncRNAs seem to play an important role in the melittin-induced suppression of PDAC cell growth and proliferation.

After identifying several hundred dysregulated lncRNAs, we focused on NONHSAT105177. We analyzed the expression of NONHSAT105177 in several pancreatic cancer cell lines and found that NONHSAT105177 expression was lower in all PDAC cell lines than in the normal pancreatic ductal cells. Interestingly, melittin treatment induced NONHSAT105177 expression in PDAC cells but not in normal HPDE. Another interesting finding is that the gain-of-function experiment confirmed the role of NONHSAT105177 in inhibiting cell proliferation and migration in vitro and in vivo. These findings suggest a crucial role for NONHSAT105177 in mediating the melittin-induced suppression of PDAC cells.

EMT is a cellular program that functions in embryogenesis, wound healing, and cancer pathogenesis by driving epithelial cells toward a mesenchymal state^12^. In cancer progression, EMT enables tumor cells to acquire highly malignant cell traits, notably the ability to disseminate and metastasize. Liu et al. reported that melittin inhibited hepatocellular carcinoma metastasis by reducing cell motility and migration^19^. We build on this work with our finding that melittin suppressed PDAC cell migration by inhibiting EMT pathway molecules (Supplementary Figure 1). We found that overexpression of NONHSAT105177 consistently reduced the expression of mesenchymal markers.

Dysregulation of cholesterol homeostasis occurs in many types of cancer and cholesterol pathway inhibition suppresses tumor growth^15,20^. Our previous study found that melittin targets the cholesterol pathway gene CLU, but the exact mechanism by which melittin regulates CLU was unknown. In this study, we hypothesized that NONHSAT105177 was associated with cholesterol pathway regulation and found that overexpression of NONHSAT105177 inhibited cholesterol pathway molecules, including CLU. Thus it is plausible that melittin downregulated CLU by inducing NONHSAT105177 expression.

We also hypothesized that melittin has a role in PDAC carcinogenesis by the trafficking of NONHSAT105177, which is mediated by exosomes. Exosomes are small membrane-encapsulated vesicles that are released into the extracellular environment by many cell types, including cancer cells. They contain a specific cargo of protein or mRNA ncRNAs, which can modulate the behavior of recipient cells^17,18,21^. Here we found that lncRNA NONHSAT105177 was transmitted between PDAC cells (Fig. 8). In other work on PDAC, Glypican-1 was found in exosomes in the context of cancer and could be used to detect early pancreatic cancer^22^. Additionally, pancreatic cancer-derived exosomes are also known to transfer microRNAs to dendritic cells and inhibit RFXAP expression via miR-212-3p^23^. This study adds to the body of work which suggests that exosomes and their cargo contribute to biological mechanisms in PDAC.

In conclusion, we found that melittin-induced suppression of PDAC cells is dependent on the lncRNA NONHSAT105177. These findings suggested that reduced expression of NONHSAT105177 may be associated with PDAC carcinogenesis. Our study has shed light on the regulatory mechanisms governed by NONHSAT105177, melittin, and CLU and indicated a potential target for developing new strategies for PDAC management.

## Materials and methods

### Cell culture

We purchased the immortalized normal human pancreatic cell line HPDE and PDAC cell lines, SW1990, Capan1, PATU8988, HS766T, BXPC3, and Panc1 from the Type Culture Collection of the Chinese Academy of Sciences (Shanghai, China). HPDE, HS766T, PATU8988, Panc1, and SW1990 cells were maintained in Dulbecco’s modified Eagle’s medium supplemented with 10% fetal bovine serum (FBS) (GIBCO, Carlsbad, CA, USA). Capan1 cells were maintained in Iscove’s modified Dulbecco’s medium containing 10% FBS. BxPC-3 cells were maintained in Roswell Park Memorial Institute (RPMI)-1640 medium containing 10% FBS. Melittin (M2272; Sigma-Aldrich, St. Louis, MO, USA) was dissolved in phosphate-buffered saline.

### RNA preparation and microarray analysis

We isolated total RNA from SW1990 cells using Trizol and treated with DNase I (Invitrogen, Carlsbad, CA, USA). Total RNA was quantified using a NanoDrop ND-2000 (Thermo Scientific, Waltham, MA, USA) and RNA integrity was assessed using an Agilent Bioanalyzer 2100 (Agilent Technologies, Santa Clara, CA, USA). The microarray chip we used in this study was Agilent Human lncRNA (4*180K, Design ID: 062918). The sample labeling, microarray hybridization, and washing were performed using the manufacturer’s standard protocols. Briefly, total RNA were transcribed to double-stranded cDNA, then synthesized into cRNA, and labeled with Cyanine-3-CTP. The labeled cRNAs were hybridized onto the microarray. After washing, the arrays were scanned with an Agilent Scanner G2505C (Agilent Technologies). We used the Feature Extraction software (version10.7.1.1, Agilent Technologies) to analyze array images and obtain raw data. We used Genespring (version 13.1, Agilent Technologies) to perform the basic analysis of the raw data. Briefly, the raw data were normalized using the quantile algorithm. The probes that at least one conditions out of two conditions have flags in “*P*” were chosen for further data analysis. Differentially expressed lncRNAs were then identified by fold change as well as their *P* value calculated using *t* test. The threshold cutoff for upregulated and downregulated lncRNAs was a fold change ≥2.0 and a *P* value ≤0.05. We identified statistically significant differentially expressed lncRNAs by volcano plot filtering. Afterward, we performed hierarchical clustering to display the distinguishable lncRNAs’ expression pattern among the samples.

### Tissue specimens

The institutional review board from the local Human Research Ethics Committee of Ruijin Hospital, Shanghai Jiao Tong University School of Medicine, China approved this study. All human participants provided informed consent. PDAC tissues and corresponding normal tissues were collected from patients who underwent pancreatectomy in the Pancreatic Disease Centre, Ruijin Hospital. None of these patients had previously received chemotherapy or radiation therapy.

### qPCR, western blot, IHC, cell proliferation assay, colony-formation assay, wound healing assay, transwell assay, and FISH

qPCR, western blot, IHC, cell proliferation assay, colony-formation assay, wound healing assay, transwell assay, and FISH were performed as described previously^10,24,25^. The PCR primers designed for genes are shown in Supplementary Table 1. The western blot experiments used antibodies to detect Snail (Cell Signaling Technology [CST], Danvers, MA, USA), Slug (CST), N-cadherin (CST), E-cadherin (CST), Vimentin (CST), β-catenin (CST), CLU (Santa Cruz Biotechnology, Dallas, TX, USA), and GAPDH (CST). The IHC experiments used antibodies to detect Ki-67 (ProteinTech, China), PCNA (ProteinTech), N-cadherin (CST), and MMP9 (ProteinTech). The sequence of the NONHSAT105177 probe for FISH was 5′-GCTCTGGATGGATTTTTACCTGGGC-3′.

### Overexpression of NONHSAT105177

Lentivirus that overexpressed NONHSAT105177 was constructed by Genomeditech (Shanghai, China). The full-length human NONHSAT105177 sequence was synthesized and subcloned into the pGMLV vector with cloning site BglII/EcoRI (Sangon, Shanghai, China). Lentivirus plasmids was transfected into HEK­293T with virus packing plasmids to produce the lentivirus. Stable expression cell lines were established according to the manufacturer’s protocol. Transfection efficiencies were evaluated by qPCR.

### Exosome experiments

We collected exosomes using standard centrifugation protocols, as previously described^26^. We used electron microscopy to examine the size and structure of exosomes. Exosomal RNA extraction was performed with the exoEasy Maxi Kit (Qiagen, Germany), according to the manufacturer’s protocol.

### Animal studies

All animal experiments were approved by the local Laboratory Animal Ethics Committee of Ruijin Hospital and conducted based on the Guide for the Care and Use Laboratory Animals of Ruijin Hospital. Animal studies were performed as described previously^27^. Briefly, 1×10^6^ PATU8988 cells were resuspended in 100 μl culture medium and injected subcutaneously into each 6-week-old male nude mouse. Mice were monitored daily for tumor growth and size, then sacrificed after 3 weeks. All tumors were harvested, weighed, and then embedded in paraffin for IHC staining.

### Statistical analysis

The experimental data were expressed as means and standard deviation and were analyzed using the SPSS16.0 software. The statistical significance of differences between the control and treated groups was determined by the paired *t* test or analysis of variance.

## Electronic supplementary material

Supplementary figure 1

Supplementary table 1

Supplementary table 2

Supplementary figure legends
