# Supplementary figures and images for: Melittin-induced long non-coding RNA NONHSAT105177 inhibits proliferation and migration of pancreatic ductal adenocarcinoma
Source: Cell Death Dis. 2018 Sep 20;9(10):940. doi: 10.1038/s41419-018-0965-3 (PMC6148000; doi:10.1038/s41419-018-0965-3)

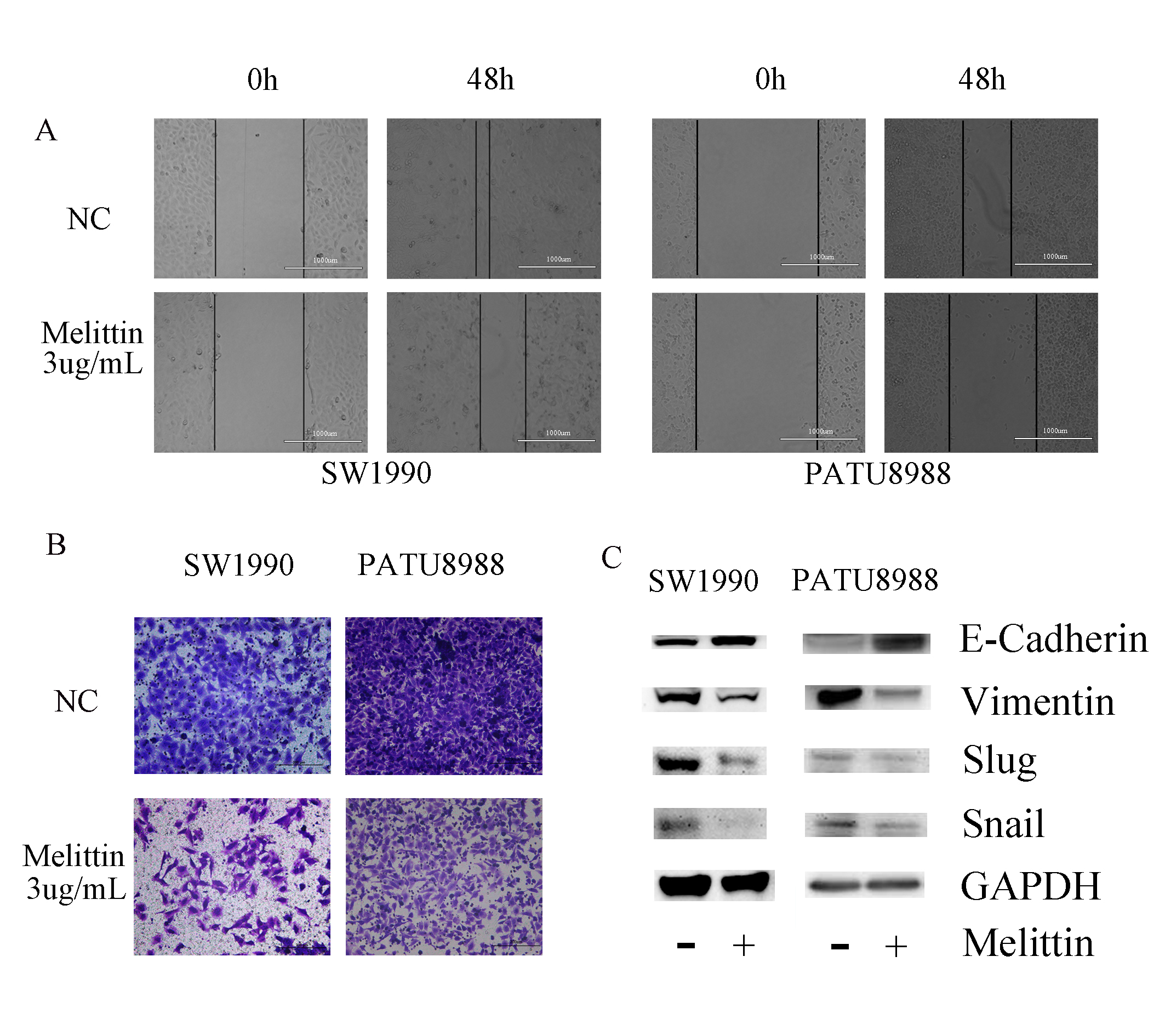

Supplement: Supplementary file 1 — Supplementary figure 1 [file 41419_2018_965_MOESM1_ESM.jpg]
