# Supplementary material for: Melittin-induced long non-coding RNA NONHSAT105177 inhibits proliferation and migration of pancreatic ductal adenocarcinoma
Source: Cell Death Dis. 2018 Sep 20;9(10):940. doi: 10.1038/s41419-018-0965-3 (PMC6148000; doi:10.1038/s41419-018-0965-3)
Supplement: Supplementary file 2 — Supplementary table 1 [file 41419_2018_965_MOESM2_ESM.docx]

Supplementary Table 1. The PCR primers designed for selected genes

| Gene name | Forward primer | Reverse primer |
| --- | --- | --- |
| ACAT2 | CTATGAGGGCTATGCCTTGCC | GCTCAGCAGTAGTAACGAAGGA |
| β-catenin | CATCTACACAGTTTGATGCTGCT | GCAGTTTTGTCAGTTCAGGGA |
| FOXC1 | TGTTCGAGTCACAGAGGATCG | ACAGTCGTAGACGAAAGCTCC |
| FOXC2 | CCTCCTGGTATCTCAACCACA | GAGGGTCGAGTTCTCAATCCC |
| Snail | ACTGCAACAAGGAATACCTCAG | GCACTGGTACTTCTTGACATCTG |
| Slug | TGTGACAAGGAATATGTGAGCC | TGAGCCCTCAGATTTGACCTG |
| TWIST1 | GTCCGCAGTCTTACGAGGAG | GCTTGAGGGTCTGAATCTTGCT |
| Vimentin | GACGCCATCAACACCGAGTT | CTTTGTCGTTGGTTAGCTGGT |
| ZEB1 | CAGCTTGATACCTGTGAATGGG | TATCTGTGGTCGTGTGGGACT |
| ZEB2 | GCGATGGTCATGCAGTCAG | CAGGTGGCAGGTCATTTTCTT |
| BMP2 | ACCCGCTGTCTTCTAGCGT | TTTCAGGCCGAACATGCTGAG |
| CLU | CTACTTCTGGATGAATGGTGACC | CGGGTGAAGAACCTGTCCT |
| CYP51A1 | ATAACCCAGCATCAGGGGAAA | CACAGTGGGAAAGTATCCATCAA |
| DHCR24 | CACTGTCTCACTACGTGTCGG | CCAGCCAATGGAGGTCAGC |
| DHCR7 | GCAGGGGTTGTGAACAAGTAT | GAGACGGCATAGCCAAGGAT |
| FDFT1 | CCACCCCGAAGAGTTCTACAA | TGCGACTGGTCTGATTGAGATA |
| HMGCR | TGATTGACCTTTCCAGAGCAAG | CTAAAATTGCCATTCCACGAGC |
| HMGCS1 | CATTAGACCGCTGCTATTCTGTC | TTCAGCAACATCCGAGCTAGA |
| HSD17B7 | TGGGATCATGCCTAATCCACA | CCAGTTCCCGAATCAGGATAAAA |
| INSIG1 | ATCCAGAGGAATGTCACTCTCTT | AGGGGTACAGTAGGCCAACAA |
| LSS | GTACGAGCCCGGAACATTCTT | CGGCGTAGCAGTAGCTCAT |
| MSMO1 | TGCTTTGGTTGTGCAGTCATT | GGATGTGCATATTCAGCTTCCA |
| SQLE | TGACAATTCTCATCTGAGGTCCA | CAGGGATACCCTTTAGCAGTTTT |
| LDLR | ACGGCGTCTCTTCCTATGACA | CCCTTGGTATCCGCAACAGA |
| ACAT2 | GCGGACCATCATAGGTTCCTT | ACTGGCTTGTCTAACAGGATTCT |
| GAPDH | ACCACAGTCCATGCCATCAC | TCCACCACCCTGTTGCTGTA |
| NONHSAT105177 | CCCTGGCTTTGGTGTTGG | GCTGGATCTCCGCTGTATTCT |
