# Supplementary material for: Melittin-induced long non-coding RNA NONHSAT105177 inhibits proliferation and migration of pancreatic ductal adenocarcinoma
Source: Cell Death Dis. 2018 Sep 20;9(10):940. doi: 10.1038/s41419-018-0965-3 (PMC6148000; doi:10.1038/s41419-018-0965-3)
Supplement: Supplementary file 4 — Supplementary figure legends [file 41419_2018_965_MOESM4_ESM.docx]

**Supplementary figure 1. Melittin prohibited pancreatic ductal adenocarcinoma (PDAC) cell growth via the epithelial-mesenchymal transition (EMT) pathway.** A. Wound healing assay with melittin treatment; B. Transwell assay with melittin treatment. C. EMT pathway protein levels (E-Cadherin, Vimentin, Snail, Slug) in response to melittin treatment. NC, negative control.
